# Lysophosphatidic acid in carcinogenesis and tumor development

**DOI:** 10.17179/excli2018-1638

**Published:** 2018-10-17

**Authors:** Wiebke Albrecht

**Affiliations:** 1 IfADo - Leibniz Research Centre for Working Environment and Human Factors at TU Dortmund, Ardeystr. 67, D-44139 Dortmund, Germany

## ⁯

Dear Editor,

Recently, Magkrioti and colleagues published a study about the autotaxin-lysophosphatidic acid axis in lung cancer (Magkrioti et al., 2018[14]). Autotaxin (ATX, ENPP2), a secreted glycoprotein that cleaves extracellular lysophosphatidylcholine to generate LPA, is the most relevant factor of extracellular lysophosphatidic acid (LPA) production. LPA is known to activate LPA receptors (LPAR), G-protein coupled receptors that show a widespread distribution and are expressed on many tumor cells (Schleicher et al., 2011[18]; Jonkers and Moolenaar, 2009[10]; Lin et al., 2010[13]; Yu et al., 2016[24]). Magkrioti et al. (2018[14]) demonstrated that a phospholipid phosphatase (LPPP3) that further metabolizes LPA was downregulated in patients with lung cancer, which may lead to increased intratumoral LPA. Moreover, genetic deletion of autotaxin as well as the LPA receptor Lpar1 attenuated tumor growth in mouse models of lung cancer (Magkrioti et al., 2018[14]).

The role of extracellular LPA in tumor development is well established (Okabe et al., 2011[17]; Leblanc et al., 2018[11]; Stuelten et al., 2018[23]; Bailey et al., 2017[1]; Fukushima et al., 2017[4]). However, recently, evidence has been presented that also intracellular LPA influences the tumor phenotype by enhancing tumor cell migration (Stewart et al., 2012[22]; Hassan, 2017[5]; Lesjak et al., 2014[12]; Marchan et al., 2012[16]). The intracellular glycerophosphocholine phosphodiesterase EDI3 (GDE5, GDPD6, or GPCPD1) hydrolyzes the glycerophosphodiester glycerophosphocholine (GPC) to glycerol-3-phosphate (G3P) and choline (Marchan et al., 2017[15]; Stewart et al., 2012[22]). G3P is further metabolized to LPA by glycerol-3-phosphate acyltransferase (GPAM). Recently, it has been shown that knockdown of GPAM decreases, while overexpression increases tumor cell migration (Marchan et al., 2017[15]). These changes in migration correspond to altered intracellular LPA concentrations (Marchan et al., 2017[15]). 

It still remains open by which mechanism intracellular LPA influences the phenotype of tumor cells. Does it act on endocytozed LPA receptors? Or are there so far unidentified interaction partners? Tumor development represents a complex process where control factors of proliferation (Schmidt et al., 2008[19]; Cadenas et al., 2014[3]; Hellwig et al., 2016[8]), interactions with the immune system (Schmidt et al., 2012[20], 2018[21]; Heimes et al., 2017[6][7]) and redox factors (Cadenas et al., 2010[2]; Jabs et al., 2017[9]) play a key role. Research how phospholipid metabolism contributes to this complex process is still at its infancy. 
